# Distinct Contribution of Global and Regional Angiotensin II Type 1a Receptor Inactivation to Amelioration of Aortopathy in *Tgfbr1*^**M318R**/+^ Mice

**DOI:** 10.3389/fcvm.2022.936142

**Published:** 2022-06-22

**Authors:** Emily E. Bramel, Rustam Bagirzadeh, Muzna Saqib, Tyler J. Creamer, Wendy A. Espinoza Camejo, LaToya Ann Roker, Jennifer Pardo Habashi, Harry C. Dietz, Elena Gallo MacFarlane

**Affiliations:** ^1^McKusick-Nathans Department of Genetic Medicine, Johns Hopkins University School of Medicine, Baltimore, MD, United States; ^2^Predoctoral Training in Human Genetics and Molecular Biology, Johns Hopkins University School of Medicine, Baltimore, MD, United States; ^3^School of Medicine Microscope Facility, Johns Hopkins University School of Medicine, Baltimore, MD, United States; ^4^Division of Pediatric Cardiology, Johns Hopkins Medicine, Baltimore, MD, United States; ^5^Howard Hughes Medical Institute, Chevy Chase, MD, United States; ^6^Department of Surgery, Johns Hopkins University School of Medicine, Baltimore, MD, United States

**Keywords:** Loeys-Dietz Syndrome, aortic aneurysm, ARBs, angiotensin II type 1 receptor, VSMC

## Abstract

Angiotensin II (Ang II) type 1 receptor (AT1R) signaling controls both physiological and pathogenetic responses in the vasculature. In mouse models of Loeys-Dietz syndrome (LDS), a hereditary disorder characterized by aggressive aortic aneurysms, treatment with angiotensin receptor blockers (ARBs) prevents aortic root dilation and associated histological alterations. In this study we use germline and conditional genetic inactivation of *Agtr1a* (coding for the AT1a receptor) to assess the effect of systemic and localized AT1R signaling attenuation on aortic disease in a mouse model of LDS (*Tgfbr1*^*M318R*/+^). Aortic diameters and histological features were examined in control and *Tgfbr1*^*M318R*/+^ mice with either germline or *Mef2C*^*SHF*^*-Cre* mediated genetic inactivation of *Agtr1a*, the latter resulting in deletion in second heart field (SHF)-derived lineages in the aortic root and proximal aorta. Both systemic and regional AT1R signaling attenuation resulted in reduction of diameters and improvement of tissue morphology in the aortic root of LDS mice; these outcomes were associated with reduced levels of Smad2/3 and ERK phosphorylation, signaling events previously linked to aortic disease in LDS. However, regional AT1a inactivation in SHF-derived lineages resulted in a more modest reduction in aortic diameters relative to the more complete effect of germline *Agtr1a* deletion, which was also associated with lower blood pressure. Our findings suggest that the therapeutic effects of AT1R antagonisms in preclinical models of aortic disease depend on both regional and systemic factors and suggest that combinatorial approaches targeting both processes may prove beneficial for aneurysm mitigation.

## Graphical Abstract

The aortic root, which is composed primarily of SHF-derived cells, is a site of increased susceptibility to dilation in both patients and mouse models of Loeys-Dietz Syndrome (*Tgfbr*1^M318R/+^). This increased risk associates with regionally increased expression of AT1a (shown in red). Both SHF-specific (AT1a-SHF^cKO^) and germline (AT1a^null^) deletion of AT1a reduce aortic root dilation and associated histopathological alterations in *Tgfbr*1^*M*318*R*/+^ mice, with the latter, however, having a more pronounced effect. 
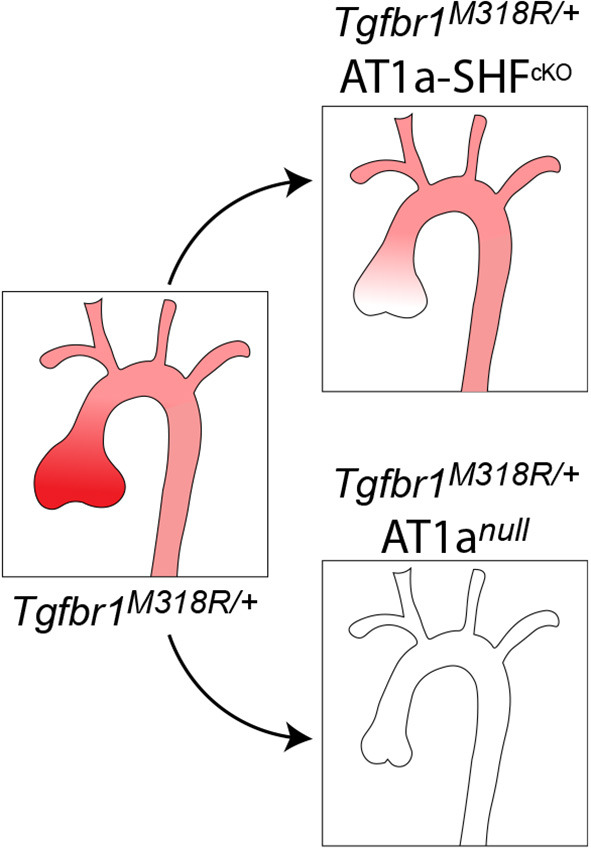

## Introduction

Aneurysms of the thoracic aorta are characterized by progressive weakening of the aortic wall resulting first in dilation and, ultimately, life-threatening dissection and rupture (1, 2). Aortic pathology is primarily linked to maladaptive changes in vascular smooth muscle cells (VSMC) and defective remodeling of the extracellular matrix; however, systemic factors, such as elevated blood pressure, can further promote disease (3). Angiotensin II (Ang II) signaling *via* the Ang II type 1 receptor (AT1R) activates several signaling pathways that can influence aneurysm pathogenesis both systemically, through regulation of vasoconstriction and fluid homeostasis, and locally, through regulation of VSMC phenotype, matrix deposition and inflammation (4–6). Although rodents have two types of AT1R, AT1a and AT1b, signaling *via* the AT1a receptor (encoded by the *Agtr1a* gene) plays the primary role in promotion of aneurysm pathogenesis (7, 8). Binding of Ang II to AT1R directly activates specific signaling cascades, including those mediated by mitogen-activated protein kinases (MAPK) (6); engagement of AT1R can also lead to transactivation of growth factor receptors and increased expression of components of other signaling pathways, including those activated by Transforming Growth Factor-β (TGF-β), Platelet-derived growth factor (PDGF), and reactive oxygen species (ROS) (5, 9–11).

Loeys-Dietz Syndrome (LDS) is a hereditary aneurysm disorder caused by heterozygous inactivating mutations in positive effectors of the TGF-β signaling pathway; these mutations result in an initial impairment of signaling output, which is followed by compensatory upregulation at sites of disease (12–19). Although these mutations occur in genes expressed ubiquitously in the aorta, the aortic root is a site of increased susceptibility to dilation (18, 19). As observed in other mouse models of aortic aneurysm, aortic root dilation in LDS mouse models is prevented by treatment with angiotensin receptor blockers (ARB), in association with lowered blood pressure and attenuated AT1R-dependent signaling in the aortic wall (5, 18–20).

Second heart field (SHF) progenitors, identified in mice by conditional genetic reporters and the *Mef2C*^*SHF*^*-Cre* transgene (21), give rise to vascular smooth muscle cells (VSMCs), aortic fibroblasts and endothelial cells, all of which contribute to morphogenesis of the aortic wall (22). Whereas SHF-derived cells predominate in the root and proximal aorta, the contribution of VSMCs derived from the cardiac-neural crest (CNC) increases progressively along the proximal-to-distal axis (23, 24). Our previous work and that of others have shown that SHF-derived VSMCs are intrinsically more sensitive to the effects of LDS-causing mutations (18, 25, 26), express higher levels of *Agtr1a*, and show increased responsiveness to Ang II in culture (18), suggesting that AT1R signaling in these cells is a contributor to pathogenesis. In this study, we test the systemic and SHF-specific contribution of AT1a receptor signaling to aortic dilation in the *Tgfbr1*^*M318R*/+^ LDS mouse model by examining the aortic phenotype in mice with either germline or *Mef2C*^*SHF*^*-Cre* mediated *Agtr1a* deletion.

## Methods

### Animals

All animal experiments were conducted following protocols approved by the Animal Care and Use Committee at Johns Hopkins University School of Medicine. Mice were housed in the animal facility with unlimited access to standard chow and water with a light/dark cycle of 10/14 h. All mice were backcrossed to 129S6/SvEv mice (Taconic, 129SVE) for at least five generations; all experiments used littermates and cohort-mates as controls. *Tgfbr1*^+/+^and *Tgfbr1*^*M*318*R*/+^(19) were bred to *Agtr1a*^*flox*/*flox*^ (The Jackson Laboratory, strain #016211) (27) mice, some bearing the *Mef2c*^*SHF*^-Cre transgene (gifted by the K.R. Chien lab at the Cardiovascular Research Center, Massachusetts General Hospital, Boston, Massachusetts, USA), to generate mice with second heart field-specific deletion of *Agtr1a*. These mice are referred to as AT1a^SHFcKO^. *Tgfbr1*^+/+^and *Tgfbr1*^*M318R*/+^ were also bred to mice with a global deletion of *Agtr1a*^*D*/*D*^, which were generated by deletion of the *Agtr1a*^*flox*^ allele *via* a germline recombination event. These mice are referred to as AT1a^null^. Mice were genotyped twice, once at the beginning and then at the end of the study, using protocols described in Chen et al. (8) for the *Agtr1a* locus and Gallo et al. (19) for the *Tgfbr1* locus.

### Echocardiography and Blood Pressure Measurements

Aortic dimensions were monitored by serial echocardiography using the Visual Sonics Vivo 2100 machine and a 30 mHz probe using a parasternal long-axis view, as previously described (18). Three independent measurements of the maximal internal diameters at the sinus of Valsalva were averaged for aortic root measurements; for ascending aorta measurements, measurements were taken at the maximal diameter. All measurements were taken during systole, with on open aortic valve. Operators blinded to genotype were responsible for imaging and measurements. Tail cuff blood pressure measurements were taken for mice using the Visitech BP-2000 Non-Invasive tail cuff device, also as previously described (19).

### RNA Extraction and qPCR

RNA was extracted according to previously described protocols (28). In brief, dissected aortic root tissue was placed in TRIzol (ThermoFisher, 15596018) and lysed using an MP Biomedicals FastPrep-24 5G automatic bead homogenizer. A Direct-zol RNA MiniPrep kit (Zymo Research, R2052) was used to extract and purify RNA according to the manufacturer's instructions. The High-Capacity cDNA Reverse Transcription kit (Applied Biosystems, 4368813) was used according to the manufacturer's protocol and qPCR was performed using TaqMan reagents and probes (Applied Biosystems, 4369016; *Agtr1a* Mm01166161_m1, *Hprt* Mm00446968_m1) and run on the QuantStuido7 Flex.

### Aortic Tissue Preparation and Histology

After euthanasia by halothane inhalation (Millipore Sigma, H0150000) at a standard concentration (4%, 0.2 ml/L of container volume), the heart and thoracic aorta were dissected *en bloc*. Samples were then fixed overnight at 4^o^C in 4% paraformaldehyde in PBS (Electron Microscopy Sciences, 15710). The following day, samples were transferred to six well plates containing 70% ethanol and left overnight at 4^o^C. The entire sample was embedded in 2% agarose prior to paraffin embedding. Paraffin blocks were then cut into 5-μm radial sections (resulting in a longitudinal view of the vessel) that were either stained with Verhoeff-van Gieson (VVG; StatLab, STVGI) or used for immunofluorescence as described below. Slides stained with VVG were imaged using an Eclipse E400 microscope (Nikon Inc.) at 40 × magnification.

### Quantification of Elastic Fiber Content

Elastic fiber content per area unit was quantified by a staff member of the Johns Hopkins School of Medicine Microscope Facility, who was blinded to the genotype of the VVG-stained sections. Color-decon2 (29) was used for unbiased automated selection of the two regions of interest (ROIs), elastic fibers and the cellular area in-between, and separation of corresponding vectors to individual channels. High intensity results, which identified blood cells, and very low intensity results, which identified non-vascular areas, were excluded from further analysis. Individual channels for each ROI were then converted to “binary” to measure the corresponding gray value (30), and this value was then converted to area to obtain the relative ratio of “elastic fiber” to “cells” content.

### Immunofluorescence

The following protocol was adapted from Cell Signaling Technology's Immunofluorescence Protocol with Formaldehyde Fixation. Paraffin-embedded sections were baked at 60°C for 15 min. Slides were deparaffinized in xylene and rehydrated by immersing in a graded alcohol series: 100% ethanol, 95% ethanol, 70% ethanol and 1 × PBS for 3 min each. Slides were then incubated in an antigen retrieval solution (10 mM sodium citrate buffer, 0.05% tween, pH 6.0) for 15 min at 90°C in a water bath. After cooling to room temperature, slides were incubated in fresh sodium borohydride solution (10 mg/ml PBS; Sigma-Aldrich, 452882) for 20 min. Slides were permeabilized with 1 × TBS (Quality Biological, 351086101) + 0.1% Triton X-100 (Sigma-Aldrich, T9284) + 0.1 M glycine (Sigma-Aldrich, G8898) for 20 min, then incubated with Fc Receptor Blocker (Innovex, NB309) for 20 min at room temperature, and then Background Buster (Innovex, NB306) for another 20 min. Slides were rinsed with 1 × TBS + Triton X-100 (TBT) and then incubated with either P-Smad3 (Abcam, ab52903) at 1:50 or P-ERK (Cell Signaling Technology, 4370) at 1:200 overnight at 4°C in a humid chamber. Slides were rinsed twice with TBT for 5 min in Donkey Anti-Rabbit Alexa Fluor 555 (ThermoFisher, A32794) at 1:100 for 45 min. Slides were again washed two times with TBT and once with TBS prior to mounting with Hard Set Mounting Media with DAPI (VECTASHIELD, H-1500). Images were acquired on a Zeiss LSM880 Airyscan FAST confocal microscope at 20 × magnification and are presented as maximal intensity projection. Image adjustments to enhance visualization of information present in the original were applied equally across samples.

### Statistics

All statistical analyses were performed using GraphPad Prism 9. A *Q* = 5% in ROUT test was selected *a priori* as an exclusion criterion for outliers. If present, outliers are shown in figures as gray circles, but not included in tests for assessment of normality, which were performed using the Shapiro-Wilk test. Data that passed normality test upon exclusion of outliers was considered normally distributed and analyzed using Brown-Forsythe and Welch ANOVA test, with no assumptions as to equal variance among groups. The two-stage linear step-up procedure of Benjamini, Krieger and Yekutieli was used for multiple comparison correction. Dataset that failed the normality test were analyzed using Kruskal–Wallis test, also followed by the two-stage linear step-up procedure of Benjamini, Krieger and Yekutieli for multiple comparison correction.

For individual time points, data are presented as a box-and-whiskers plot, with whiskers indicating minimum to maximum points, with all points shown. Growth curves over time were compared using a linear regression model, with least-square regression and no weighting; comparison between slopes was performed using the extra-sum-of-squares *F*-test, with *P* = 0.05. Error bars in growth curve plots refer to the 95% confidence interval (CI). Survival tables were analyzed using Fisher's exact test.

## Results

*Tgfbr1*^*M318R*/+^ mice (also referred to as LDS mice in this text), recapitulate many of the features observed in LDS patients, including dilation of the aortic root (18, 19). To determine if global or SHF-specific attenuation of AT1R signaling mitigated aortic dilation in LDS mice, we crossed these and control mice to either mice homozygous for the *Agtr1a*^*D*^ allele (*Agtr1a*^*D*/*D*^, referred to as AT1a^null^) or to *Agtr1a*^*flox*/*flox*^ mice also expressing the *Mef2c*^*SHF*^*-Cre* (21) transgene. Serial echocardiography was performed from 8 to 24 weeks of age, and aortic tissue collected and processed for histological analysis at the 24-week timepoint as previously described (18, 19). Blood pressure was also measured prior to sacrifice. Analysis of an initial experimental cohort showed that there were no significant differences in aortic measurements or rate of aortic enlargement between male and female *Tgfbr1*^*M318R*/+^ mice (Supplementary Figures 1A–C) however, control male mice were significantly larger than their female counterparts, and the difference in aortic diameters between female and male *Tgfbr1*^*M318R*/+^ mice at the 24-week time point approached significance (*P* = 0.06; Supplementary Figure 1B). For these reasons, male and female mice were analyzed separately according to current guidelines.

We found that the presence of the *Mef2c*^*SHF*^*-Cre* transgene in mice also carrying a *Agtr1a*^*flox*^allele resulted in relatively frequent recombination in the germline, leading to generation of both *Agtr1a*^*flox*/*D*^*; Mef2c*^*SHF*^*-Cre* and *Agtr1a*^*flox*/*flox*^*; Mef2c*^*SHF*^*-Cre* litters when breeding *Agtr*1*a*^*flox*/^+; *Mef2c*^*SHF*^*-Cre* or *Agtr*1*a*^*flox*/^flox *Mef2c*^*SHF*^*-Cre* mice. Germline recombination occurred in both male and female breeders whenever the *Agtr1a*^*flox*^allele was present in mice also carrying the *Mef2c*^*SHF*^*-Cre* transgene. However, the presence of the *Agtr1a*^*D*^ null allele in heterozygosity did not significantly affect blood pressure, *Agtr1a* expression in the aorta, nor aortic size in either *Tgfbr1*^+/+^or *Tgfbr1*^*M318R*/+^ mice (Supplementary Figures 2, 3). Therefore, *Agtr1a*^*flox*/*D*^ and *Agtr1a*^*flox*/*flox*^are collectively referred to as controls (AT1a^Ctrl^) in the absence of the *Cre* recombinase, and as AT1a SHF-deficient mice (AT1a^SHFcKO^) if also expressing the *Mef2c*^*SHF*^*-Cre* recombinase. All ultrasound measurements and genotypes are provided in Supplementary Table 1.

**Figure 1:**
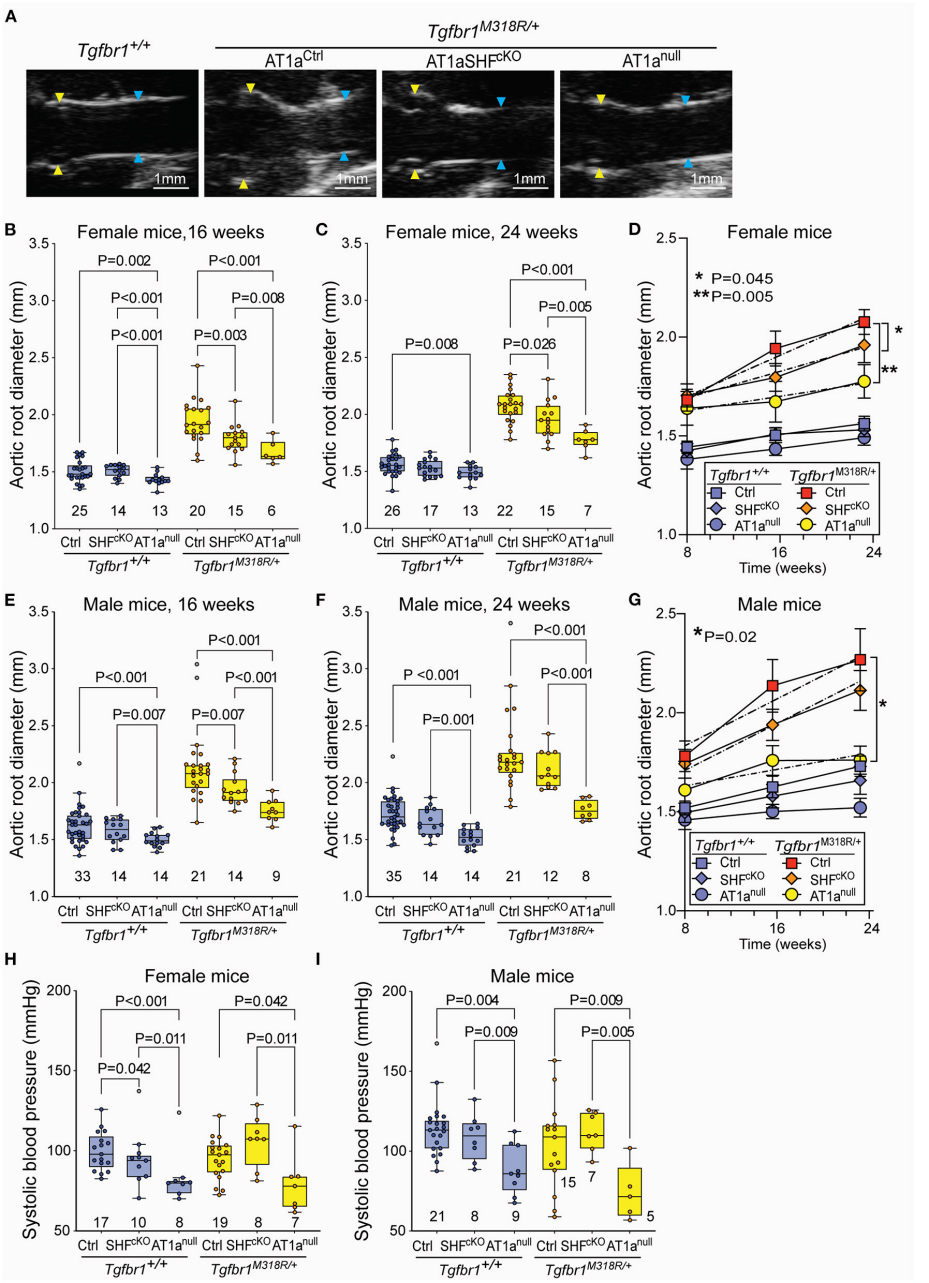
Global and SHF-specific deletion of *Agtr1a* result in reduction of aortic root dilation in LDS mice. **(A)** Representative echocardiograms of *Tgfbr1*^+/+^ and *Tgfbr1*^*M318R*/+^mice with and without conditional (AT1a^SHFcKO^) or global (AT1a^null^) deletion of *Agtr1a*, the aortic root is indicated by yellow arrows while the ascending aorta is indicated by blue arrows. Aortic root diameter as measured by echocardiography in both females **(B–D)** and males **(E–G)** at indicated time points. *Tgfbr1*^+/+^ mice are shown in blue and *Tgfbr1*^*M318R*/+^are shown in yellow. The number of animals per group is indicated. *P*-values refer to Brown-Forsythe ANOVA, followed by *post-hoc* test with multiple comparison FDR correction. In panel **(C,E)**, the error bars represent the 95% Confidence Interval (CI), the dashed line indicates a simple linear-regression of serial echocardiographic measurements of aortic root diameter from 8 to 24 weeks of age; *P*-value refers to comparison between slopes using the extra-sum-of-squares *F*-test in GraphPad. **(H,I)** Systolic blood pressure as measured at 24 weeks of age. *P*-values refer to Brown-Forsythe ANOVA, followed by *post-hoc* test with multiple comparison FDR correction.

Homozygous deletion of *Agtr1a* in SHF-derived lineages resulted in reduced aortic root dimeters in both female and male *Tgfbr1*^*M318R*/+^ mice relative to AT1a^Ctrl^ controls at the 16-week time-point; however, this effect remained significant only in female mice by 24 weeks of age (Figures 1A–G). Female AT1a^SHFcKO^ *Tgfbr1*^*M318R*/+^ mice but not male mice also showed a reduced rate of growth from 8 to 24 weeks relative to AT1a^Ctrl^ *Tgfbr1*^*M318R*/+^ mice (Figures 1D,G). Blood pressure was not significantly affected by deletion of *Agtr1a* in SHF-derived lineages (Figures 1H,I).

Homozygous germline *Agtr1a* deletion resulted in reduction of aortic root diameters and rate of growth in both male and female LDS mice up to 24 weeks of age (Figures 1A–G), and also associated with a reduction in blood pressure, consistent with previous analyses of AT1a^null^ mice (31) (Figures 1H,I). No significant differences were observed in the diameter of the ascending aorta (Supplementary Figure 4A). Although our study was not designed nor powered to detect differences in survival, a significant decrease in survival between male *Tgfbr1*^*M318R*/+^ relative to control *Tgfbr1*^+/+^ mice was noted (Supplementary Figure 4B).

**Figure 2:**
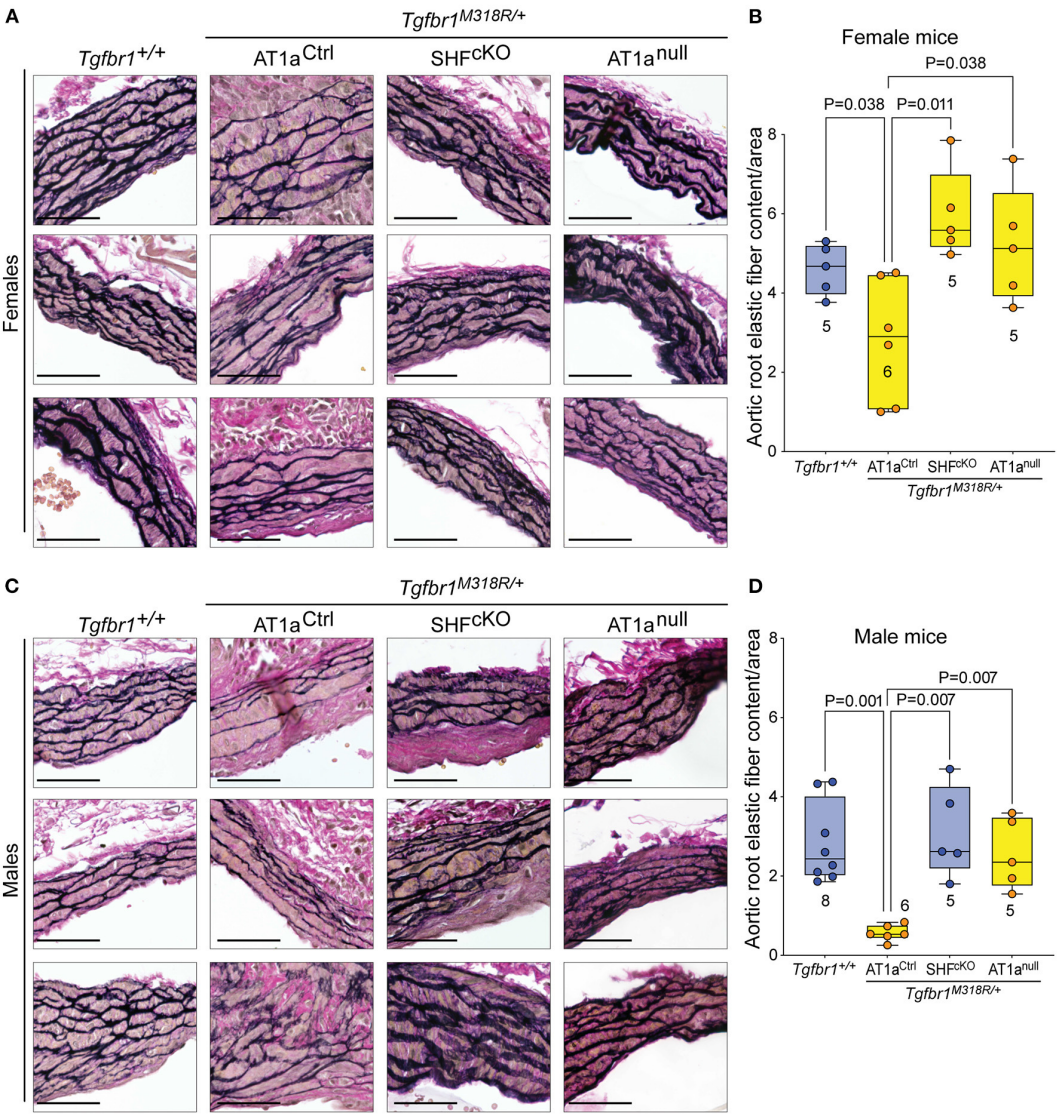
Global and SHF-specific deletion of *Agtr1a* result in improved histopathology. Representative sections of VVG-stained aortic roots and quantification of elastic fiber content relative to cellular area for female **(A,B)** and male **(C,D)** samples of indicated genotypes. Three representative images are shown per genotype for each sex. Images are shown at 20 × magnification. Scale bar is 50 μm. Quantification of elastic fiber content relative to cellular area is shown in **(C)** for female samples, and in **(D)** for male samples, with higher value indicating a greater content of elastic fiber per area unit. The number of mice scored per group is indicated. *P*-values refer to Brown-Forsythe ANOVA, followed by *post-hoc* test with multiple comparison FDR correction.

**Figure 3:**
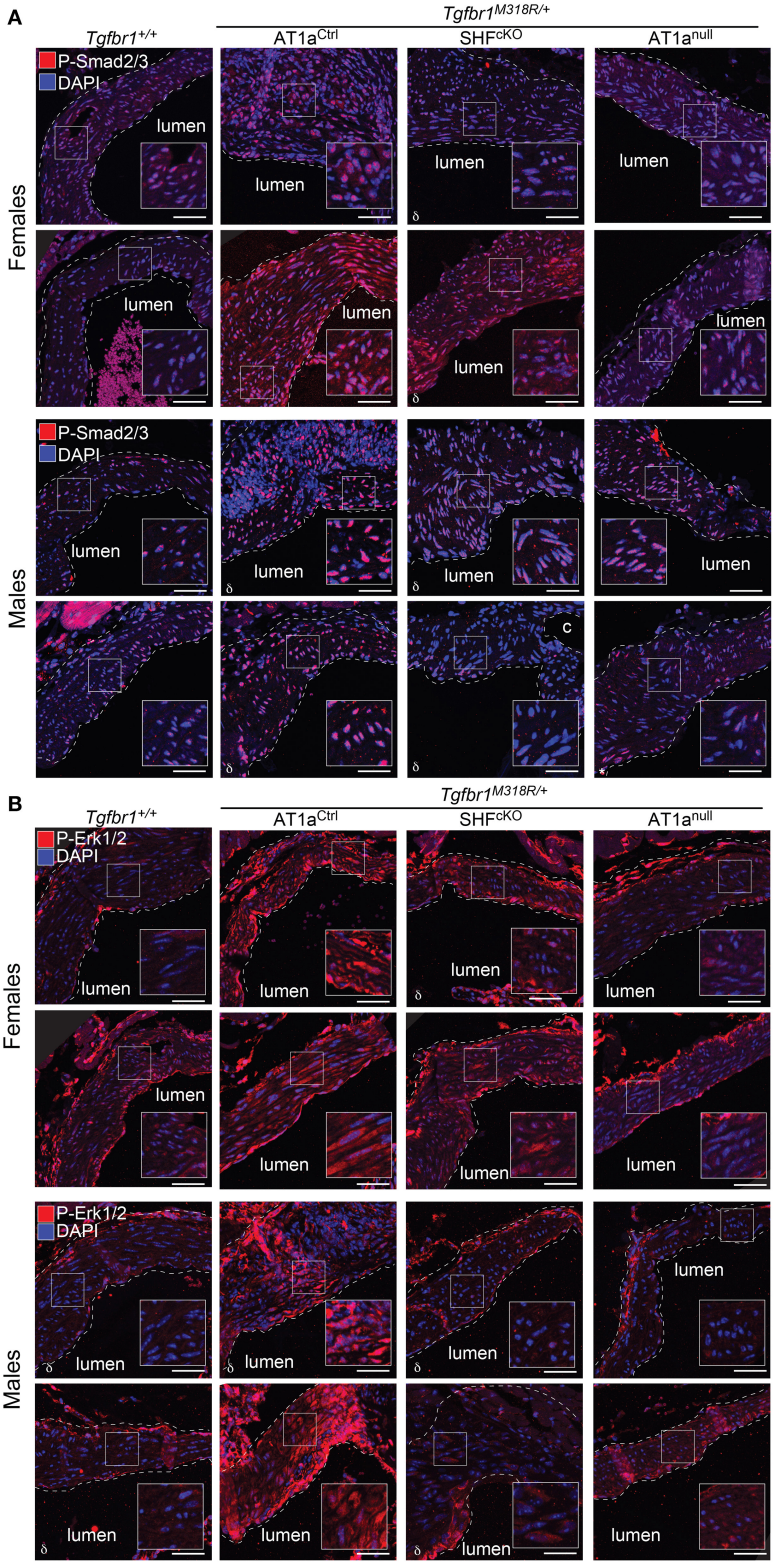
Global and SHF-specific deletion of *Agtr1a* result in reduced p-Smad2/3 and p-ERK1/2 signaling. Representative images of immunofluorescence for **(A)** p-Smad2/3 and **(B)** p-ERK1/2 at 24 weeks of age. Four independent biological replicates are shown per genotype, two from male animals and two from female animals. Insets identify locations shown at higher magnification. Images were acquired at 20 × magnification. Scale bar is 50 μm. δ symbol in the left-hand corner of a given image indicates the section is from an animal heterozygous for the *Agtr1a*^*D*^ null allele.

Histological sections of aortas were stained to visualize tissue architecture and elastic fibers, and the relative content of elastic fiber to cellular area was quantified using an ImageJ macro. Both AT1a^null^ and AT1a^SHFcKO^ *Tgfbr1*^*M318R*/+^ mice, of either sex, showed improved elastic fiber content relative to AT1a^Ctrl^ *Tgfbr1*^*M318R*/+^ mice (Figure 2 and Supplementary Figure 5). This improvement correlated with reduced levels of phosphorylation of both Smad2 and Smad3 (p-Smad2/3) and extracellular signal-regulated kinase 1 and 2 (p-ERK1/2), two signaling events previously shown to correlate with severity of aortic disease in mouse models of LDS and related conditions (5) (Figures 3A,B and Supplementary Figure 6). No specific localization relative to inner or outer media was noted for either p-Smad2/3 or p-ERK1/2 signal, possibly in consequence of the advanced stage of aortic disease and media disruption at the time point examined. However, whereas p-ERK1/2 signal was detectable in the endothelial and adventitial layer regardless of genotype or disease status, both systemic and SHF-specific AT1a deletion resulted in reduced p-ERK1/2 levels across the media compared *Tgfbr1*^*M318R*/+^ mice, which is consistent with previous observations (19).

## Discussion

Administration of ARBs such as losartan has been shown to ameliorate aortic pathology in several mouse models of aneurysm, including LDS (5, 8, 18–20, 32). However, experiments based on pharmacological antagonism cannot disentangle the potential benefits of local antagonism from those accrued thanks to systemic effects. In addition, the existence of potential off-targets for drugs such as losartan has led to the hypothesis that some benefits of ARB administration may result from AT1R-independent effects (33).

In this study, we show that genetic deletion of AT1a in SHF-derived cells, which include VSMC residing primarily in the aortic root but also fibroblasts and subsets of endothelial cells (22), mitigates aortic dilation and improves aortic tissue architecture in LDS mice. However, this intervention fails to recapitulate the more robust reduction in aortic diameters observed after germline AT1a inactivation, particularly at later time points in male mice. We hypothesize that the additional benefit of systemic AT1a inactivation on aortic diameters may be due to both ablation in AT1a receptor signaling in non-SHF-derived cells and, possibly, lessening of mechanical stresses on the weakened wall secondary to reduction in blood pressure. Although our previous work showed that only SHF-derived VSMCs had higher expression of *Agtr1a* and increased responsiveness to Ang II (18), it is possible that AT1a receptor signaling in non-SHF-derived cells, including endothelial cells, may also play a role in LDS aortic dilation, similarly to what has been observed in other mouse models (27, 34).

Although we did not observe significant sexual dimorphism in the absence of additional genetic perturbations, there was a trend for larger aortic root diameters in male *Tgfbr1*^*M318R*/+^ mice relative to their female counterparts, especially at later time points. This was accompanied by a significant reduction of aortic root diameters in female but not male *Tgfbr1*^*M318R*/+^ mice with homozygous *Agtr1a* deletion in SHF-derived cells relative to controls. The presence of sexual dimorphism in LDS mouse models under specific circumstances is consistent with reports showing similar patterns in other hereditary connective tissue disorders, including both patients and mouse models of Marfan and Ehlers-Danlos syndrome (35–38). Although the mechanisms remain unclear, angiotensin II-driven aortic pathogenesis has been consistently shown to be accentuated in males relatively to females (39). We hypothesize that the severity of aortic disease in LDS mouse models generally masks the subtle effect of sexually dimorphic hormonal or chromosomal contributions, and that these effects may become more apparent in specific contexts that result in moderate amelioration of pathogenesis.

Despite its well-documented efficacy in preclinical animal models, randomized trials using the ARB losartan at doses sufficient to successfully reduced blood pressure have shown more modest and sometimes mixed results in the treatment of aneurysm in Marfan syndrome (MFS) (5, 40–42). Our study suggests that regional AT1R inhibition may be important for amelioration of aortic tissue architecture, and that better outcomes may be possible using strategies able to suppress both local and systemic AT1R signaling. Although this study explored the effects of AT1R inhibition only in the proximal thoracic aorta, the multiplicity of roles played by this signaling pathway in both physiological and pathological processes suggests that its inhibition may influence other LDS-associated phenotypes (12–19). An exploration of the effects of AT1 inhibition on both vascular and non-vascular LDS connective tissue anomalies would provide stronger evidence of therapeutic benefit.

Notably, a recent study by Chen et al. (8) has shown that administration of antisense oligonucleotides results in stable and durable reduction in levels of angiotensinogen, the precursor to Ang II, resulting in protection from aortic disease in the *Fbn1*^*C*1041*G*/+^ MFS mouse model. We speculate strategies that better mimic the early, robust, and continuous suppression of AT1R signaling achieved in animal models by germline deletion of *Agtr1a* (or by early ARB administration in drinking water and/or osmotic pump) may prove more efficacious for medical therapy in LDS and related conditions.

## Data Availability Statement

The original contributions presented in the study are included in the article/Supplementary Material, further inquiries can be directed to the corresponding author.

## Ethics Statement

The animal study was reviewed and approved by the Johns Hopkins University Animal Care and Use Committee.

## Author Contributions

EG and HD are responsible for the conception and design of this study. RB initiated much of the experimental work, including animal breeding and echocardiography. JP acquired and analyzed echocardiographic images. EB and TC assisted with echocardiography and animal breeding. MS assisted in genotyping and performed the histological staining. EB and WE performed the immunofluorescence. LR developed a macro in Image J to quantify the elastic fiber content relative to cellular area in aortic sections. EB assisted EG in writing and preparing figures for the manuscript. All authors contributed to manuscript revision, read, and approved the submitted version.

## Funding

Research reported in this publication was supported by the National Heart, Lung, and Blood Institute of the National Institutes of Health under award number R01HL147947 and by a generous gift from the Loeys-Dietz Foundation. EM was also supported by funding provided to Johns Hopkins by the Broccoli family. Image acquisition was also supported by NIH award number S10OD023548 to the School of Medicine Microscope Facility.

## Conflict of Interest

The authors declare that the research was conducted in the absence of any commercial or financial relationships that could be construed as a potential conflict of interest.

## Publisher's Note

All claims expressed in this article are solely those of the authors and do not necessarily represent those of their affiliated organizations, or those of the publisher, the editors and the reviewers. Any product that may be evaluated in this article, or claim that may be made by its manufacturer, is not guaranteed or endorsed by the publisher.
