# Supplementary material for: Distinct Contribution of Global and Regional Angiotensin II Type 1a Receptor Inactivation to Amelioration of Aortopathy in Tgfbr1M318R/+ Mice
Source: Front Cardiovasc Med. 2022 Jun 22;9:936142. doi: 10.3389/fcvm.2022.936142 (PMC9257222; doi:10.3389/fcvm.2022.936142)
Supplement: Supplementary file 1 [file Data_Sheet_1.PDF]

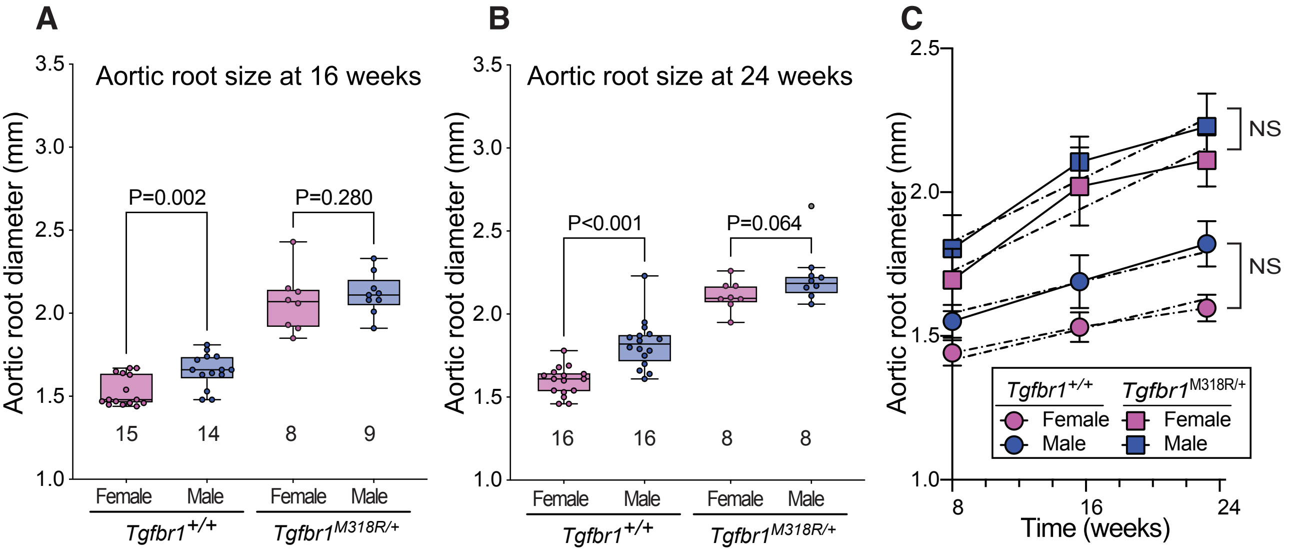

Supplemental Figure 1. LDS *Tgfbr1$^{M318R/+}$* mice do not show overt sexual dimorphism. Aortic root diameter of female and male *Tgfbr1$^{M318R/+}$* and control mice at 16 weeks (**A**) and 24 weeks (**B**) of age, as measured by echocardiography. The number of animals per group is indicated. P-values refer to Brown-Forsythe ANOVA, followed by post-hoc test with multiple comparison FDR correction. (**C**) Growth curves for mice of indicated genotype. Error bars represent the 95% Confidence Interval (CI), the dashed line indicates a simple linear-regression of serial echocardiographic measurements of aortic root diameter from 8-24 weeks of age; P-value refers to comparison between slopes using the extra-sum-of-squares F test in GraphPad.
