# Supplementary material for: Distinct Contribution of Global and Regional Angiotensin II Type 1a Receptor Inactivation to Amelioration of Aortopathy in Tgfbr1M318R/+ Mice
Source: Front Cardiovasc Med. 2022 Jun 22;9:936142. doi: 10.3389/fcvm.2022.936142 (PMC9257222; doi:10.3389/fcvm.2022.936142)
Supplement: Supplementary file 2 [file Data_Sheet_2.PDF]

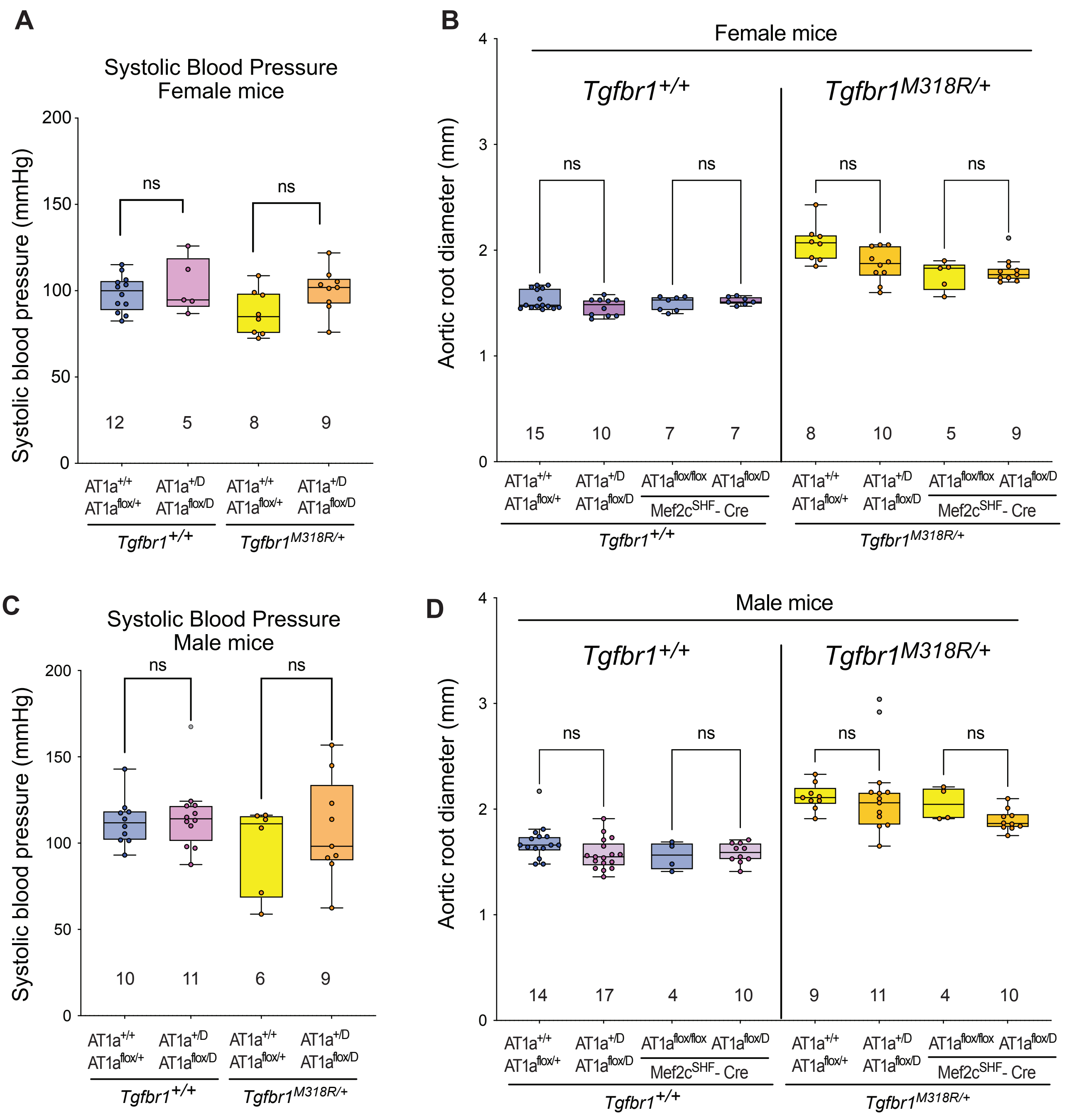

Supplemental Figure 2. Mice carrying one null *Agtr1a* allele show no significant alterations in blood pressure or aortic root diameter relative to matched controls. Systolic blood pressures and aortic root diameters (16 weeks of age) in female mice (**A, B**) and male mice (**C, D**) grouped by presence or absence of one null *Agtr1a*^{D} allele. P-values refer to Brown-Forsythe ANOVA (**A**, **B**), or Kruskal-Wallis test (**C, D**), followed by post-hoc test with multiple comparison FDR correction. Blue indicates measurements for control animals with no *Agtr1a*^{D} allele, while purple indicates measurements for control animals with one *Agtr1a*^{D} null allele. Yellow indicates measurements for *Tgfbr1*$^{M318R/+}$ animals with no *Agtr1a*^{D} allele, while orange indicates measurements for *Tgfbr1*$^{M318R/+}$ with one *Agtr1a*^{D} null allele. Whiskers in the box and whisker plot indicate the maximum-to-minimum range.
