# Supplementary material for: Distinct Contribution of Global and Regional Angiotensin II Type 1a Receptor Inactivation to Amelioration of Aortopathy in Tgfbr1M318R/+ Mice
Source: Front Cardiovasc Med. 2022 Jun 22;9:936142. doi: 10.3389/fcvm.2022.936142 (PMC9257222; doi:10.3389/fcvm.2022.936142)
Supplement: Supplementary file 3 [file Data_Sheet_3.PDF]

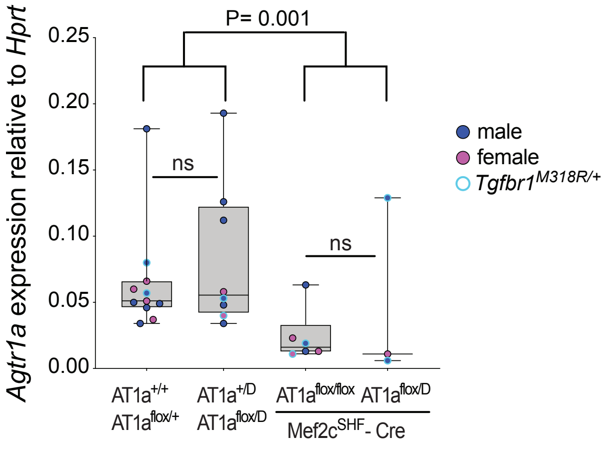

Supplemental Figure 3. Aortic root samples from AT1a^{SHFcKO} mice show significantly lower *Agtr1a* mRNA expression relative to controls. qPCR analysis of *Agtr1a* relative to *Hprt* expression in aortic root samples from control and AT1a^{SHFcKO} animals with and without the presence of one *Agtr1a^{D}* null allele. All individual data points are shown, purple data points are from female animals while blue are from male animals. Data points with a light blue outline are from *Tgfbr1$^{M318R/+}$* mice. Whiskers in the box and whisker plot indicate the maximum-to-minimum range. P-value refers to a two-tailed Mann-Whitney test.
