# Supplementary material for: Distinct Contribution of Global and Regional Angiotensin II Type 1a Receptor Inactivation to Amelioration of Aortopathy in Tgfbr1M318R/+ Mice
Source: Front Cardiovasc Med. 2022 Jun 22;9:936142. doi: 10.3389/fcvm.2022.936142 (PMC9257222; doi:10.3389/fcvm.2022.936142)
Supplement: Supplementary file 4 [file Data_Sheet_4.PDF]

A

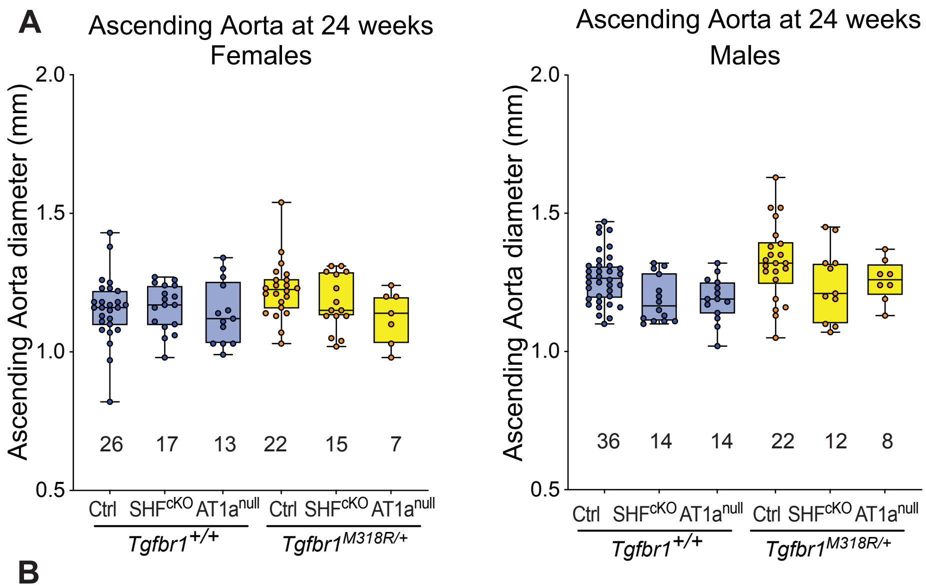

B

FEMALES	*Tgfbr1*$^{+/+}$			*Tgfbr1*$^{M318R/+}$		
8-24 weeks	AT1a^{Ctrl}	AT1a^{SHFcKO}	AT1a^{null}	AT1a^{Ctrl}	AT1a^{SHFcKO}	AT1a^{null}
Live	26	17	14	24	15	7
Dead	0	0	0	3	0	0
Total	26	17	14	27	15	7

MALES	*Tgfbr1*$^{+/+}$			*Tgfbr1*$^{M318R/+}$		
8-24 weeks	AT1a^{Ctrl}	AT1a^{SHFcKO}	AT1a^{null}	AT1a^{Ctrl}	AT1a^{SHFcKO}	AT1a^{null}
Live	36	14	14	22	12	9
Dead	0	0	0	6*	4	1
Total	36	14	14	28	16	10

* Fisher's exact test, P=0.005 between control *Tgfbr1*$^{+/+}$ and *Tgfbr1*$^{M318R/+}$

Supplemental Figure 4. No significant differences in ascending aortic diameter or survival across tested genetic alterations of LDS mice. (A) Ascending aorta diameter of *Tgfbr1*$^{+/+}$ and *Tgfbr1*$^{M318R/+}$ mice with and without conditional (AT1a^{SHFcKO}) or global (AT1a^{null}) homozygous deletion of *Agtr1a* at 24 weeks of age as measured by echocardiography. P-values refer to Brown-Forsythe ANOVA, followed by post-hoc test with multiple comparison FDR correction. **(B)** Total number of mice per group reported as alive or dead during the 8 to 24 weeks monitoring period. P-value refers to Fisher's exact test.
