# Supplementary material for: Distinct Contribution of Global and Regional Angiotensin II Type 1a Receptor Inactivation to Amelioration of Aortopathy in Tgfbr1M318R/+ Mice
Source: Front Cardiovasc Med. 2022 Jun 22;9:936142. doi: 10.3389/fcvm.2022.936142 (PMC9257222; doi:10.3389/fcvm.2022.936142)
Supplement: Supplementary file 5 [file Data_Sheet_5.PDF]

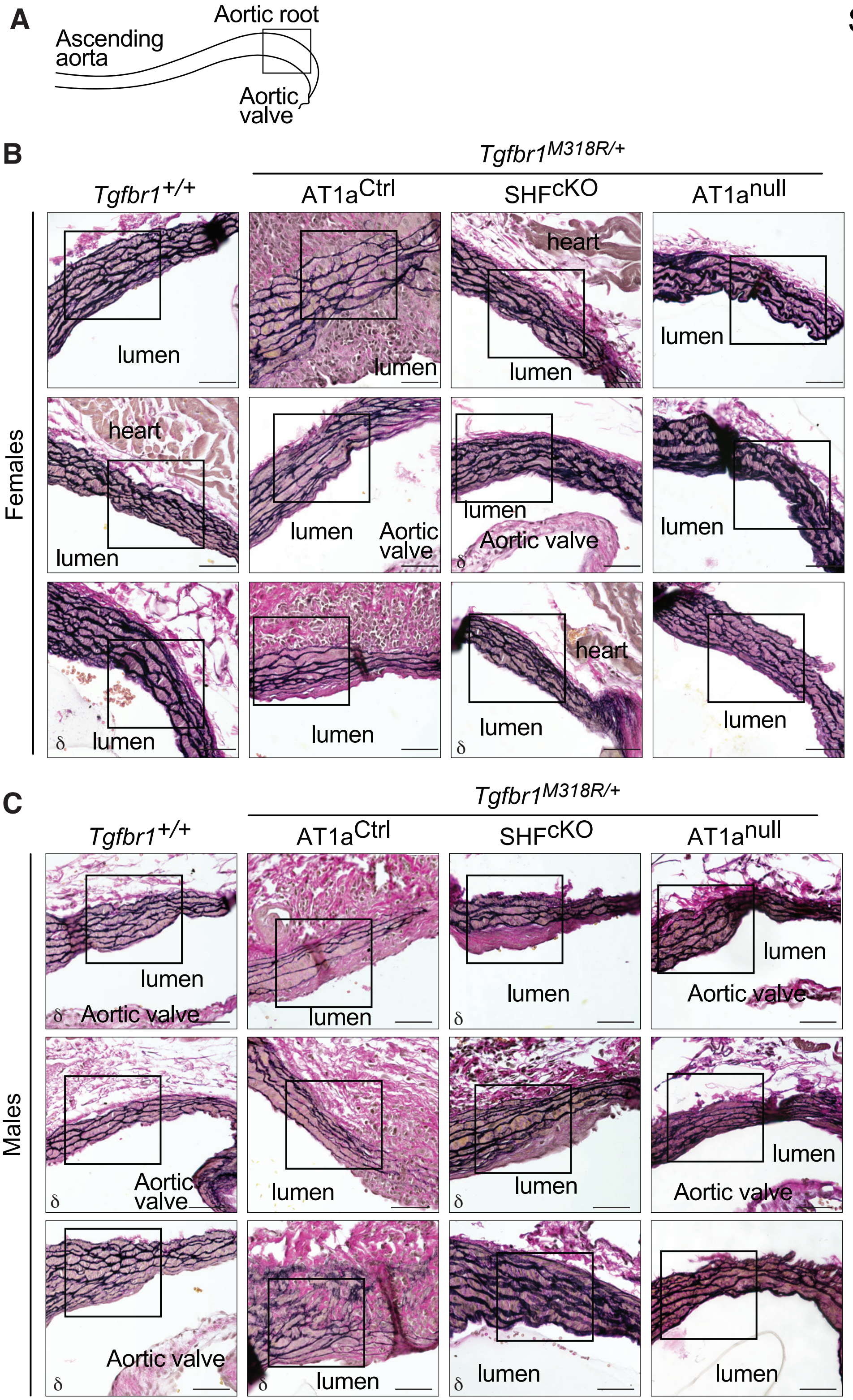

Supplemental Figure 5. VVG-stained radial sections of the proximal thoracic aorta. (A) Diagram indicating location of aortic sections shown. VVG staining was performed on radial sections to create a longitudinal view of the proximal thoracic aorta. Representative sections of VVG-stained aortic roots obtained from female (**B**) and male (**C**) *Tgfbr1*$^{+/+}$ and *Tgfbr1*$^{M318R/+}$ mice with and without conditional (AT1a^{SHFcKO}) or global (AT1a^{null}) at 40x magnification. Scale bar is 100µm. δ symbol in the left-hand corner of a given image indicates the section is from an animal with one *Agtr1a*^{D} null allele. Boxes indicate the area shown at higher magnification in Figure 2.
