# Supplementary material for: Distinct Contribution of Global and Regional Angiotensin II Type 1a Receptor Inactivation to Amelioration of Aortopathy in Tgfbr1M318R/+ Mice
Source: Front Cardiovasc Med. 2022 Jun 22;9:936142. doi: 10.3389/fcvm.2022.936142 (PMC9257222; doi:10.3389/fcvm.2022.936142)
Supplement: Supplementary file 6 [file Data_Sheet_6.PDF]

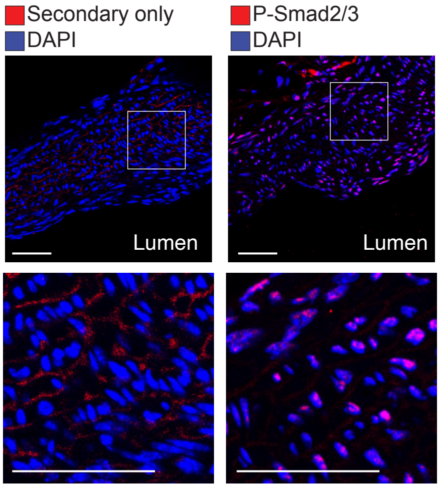

Supplemental Figure 6. Positive and negative control for immunofluorescence in thoracic aorta with Alexa Fluor 555. Immunofluorescence negative control performed using only Alexa Fluor 555 secondary and no primary antibody and a positive control using P-Smad2/3 primary antibody and Alexa Fluor 555 secondary. Insets identify locations shown at higher magnification. Images were acquired at 20x magnification. Scale bar is 50μm.
